# Accurate Localization of the Internal Opening in a Cryptoglandular Anal Fistula: Complementary Roles of Goodsall’s Rule, MRI, and Intraoperative Assessment: A Case Report

**DOI:** 10.1155/cris/3016034

**Published:** 2026-10-07

**Authors:** Võ Đắc Truyền

**Affiliations:** ^1^ Faculty of Medicine Dong A University Da Nang Vietnam

**Keywords:** anal fistula, cryptoglandular fistula, Goodsall’s rule, internal opening, magnetic resonance imaging

## Abstract

Accurate localization of the internal opening is essential for successful surgical management of cryptoglandular anal fistulas. Although Goodsall’s rule remains a useful clinical guide, accurate delineation of fistula anatomy often requires complementary assessment with magnetic resonance imaging (MRI) and intraoperative evaluation. We report the case of a 34‐year‐old healthy man presenting with a recurrent perianal fistula characterized by intermittent swelling and purulent discharge. The external opening was located at the 2 o’clock position, more than 3 cm from the anal verge. Preoperative MRI demonstrated a curved transsphincteric fistulous tract extending to an internal opening at the posterior midline without secondary extensions or abscess. The internal opening was confirmed intraoperatively by clinical assessment and hydrogen peroxide injection through the external opening. The extrasphincteric portion of the tract and surrounding chronically inflamed tissue were excised (partial fistulectomy), whereas the transsphincteric portion crossing the external anal sphincter was treated by lay‐open fistulotomy to minimize additional sphincter tissue sacrifice. The postoperative course was uneventful, with complete wound healing within 2 months and no impairment of continence. This case illustrates that accurate localization of the internal opening is best achieved through the complementary use of clinical assessment, Goodsall’s rule, MRI, and intraoperative confirmation, thereby facilitating appropriate surgical planning and sphincter‐preserving treatment.

## 1. Introduction

Anal fistula is a common anorectal disorder in which successful surgical treatment depends primarily on accurate identification of the fistulous tract and, most importantly, the internal opening. Failure to identify the internal opening may result in persistent sepsis, recurrence, or unnecessary division of the anal sphincter, thereby increasing the risk of postoperative fecal incontinence [1, 2].

Before the widespread availability of modern imaging techniques, Goodsall’s rule served for more than a century as a simple and practical clinical guide for predicting the location of the internal opening and the likely course of the primary fistulous tract according to the position of the external opening [3, 4]. Although the rule remains useful during clinical examination, subsequent studies have demonstrated that its predictive accuracy is imperfect, particularly in long anterior fistulas and in cases where the fistula anatomy does not conform to the expected pattern [5–8].

Magnetic resonance imaging (MRI) is currently regarded as the reference imaging modality for the preoperative assessment of anal fistulas because it accurately delineates the primary tract, identifies the internal opening, evaluates sphincter involvement, and detects secondary extensions or occult abscesses that may influence surgical planning [2, 9–11]. Consequently, MRI has become an essential investigation in patients with suspected complex fistulas or when the anatomy cannot be confidently defined by clinical examination alone [2, 11].

We report a case in which the external opening was located anteriorly and more than 3 cm from the anal verge, whereas contrast‐enhanced MRI demonstrated a posterior midline internal opening. This case illustrates that accurate localization of the internal opening is best achieved through the complementary use of clinical assessment, Goodsall’s rule, MRI, and intraoperative confirmation, thereby facilitating appropriate surgical planning and sphincter‐preserving treatment.

## 2. Case Presentation

A 34‐year‐old otherwise healthy man presented with an 8‐month history of recurrent swelling and intermittent purulent discharge from the left perianal region. He denied fever, weight loss, inflammatory bowel disease, previous anorectal surgery, or any significant medical history.

Physical examination revealed a single external opening located at the 2 o’clock position, more than 3 cm from the anal verge, with minimal surrounding inflammation and a small amount of mucopurulent discharge (Figure 1). No fluctuation or associated abscess was detected. Digital rectal examination demonstrated normal resting anal sphincter tone without palpable intraluminal masses.

**Figure 1:**
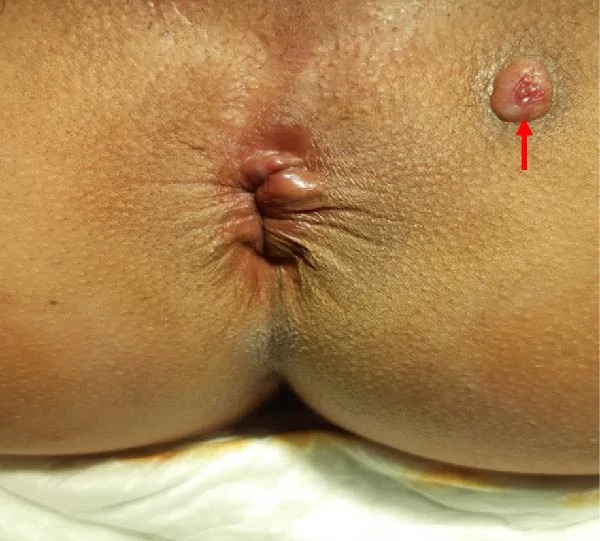
Clinical photograph demonstrating the external opening (red arrow) at the 2 o’clock position, located more than 3 cm from the anal verge.

Routine laboratory investigations, including complete blood count, coagulation profile, serum biochemistry, chest radiography, and electrocardiography, were unremarkable.

Pelvic MRI demonstrated a single transsphincteric fistulous tract arising from the anterior external opening (Figure 2), coursing posteriorly toward the posterior midline internal opening located at the level of the dentate line (Figure 3A,B), without extension above the anorectal ring. Coronal contrast‐enhanced MRI further demonstrated the curved primary tract without secondary extensions or associated abscess (Figure 4). MRI also showed that the main fistulous tract traversed ~30% of the external anal sphincter thickness, which measured 11.23 mm (Figure 5).

**Figure 2:**
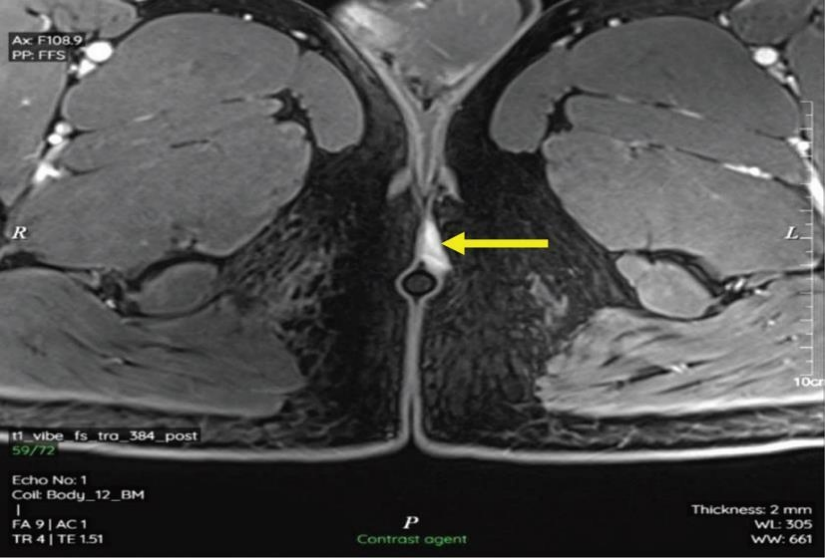
Axial gadolinium‐enhanced T1‐weighted MRI demonstrating the anterior external opening (yellow arrow).

**Figure 3:**
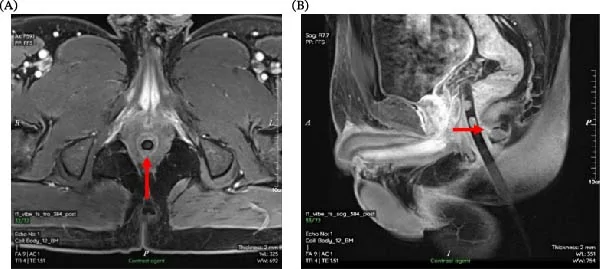
MRI demonstrating the internal opening. (A) Axial gadolinium‐enhanced T1‐weighted image showing the internal opening at the posterior midline (red arrow). (B) Sagittal gadolinium‐enhanced T1‐weighted image confirming the posterior midline location of the internal opening (red arrow).

**Figure 4:**
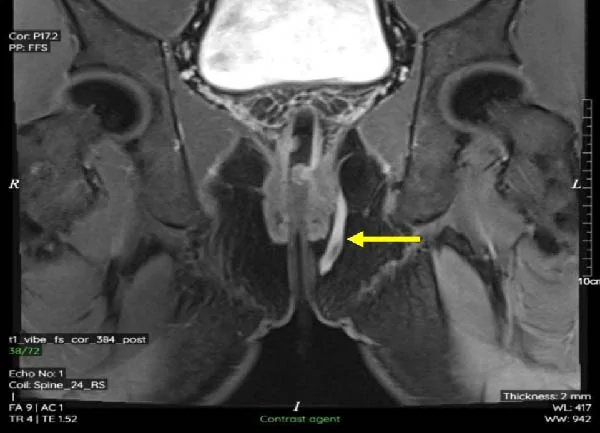
Coronal contrast‐enhanced MRI demonstrating the primary transsphincteric fistulous tract (yellow arrow) curving from the external opening at the 2 o’clock position toward the internal opening at the posterior midline.

**Figure 5:**
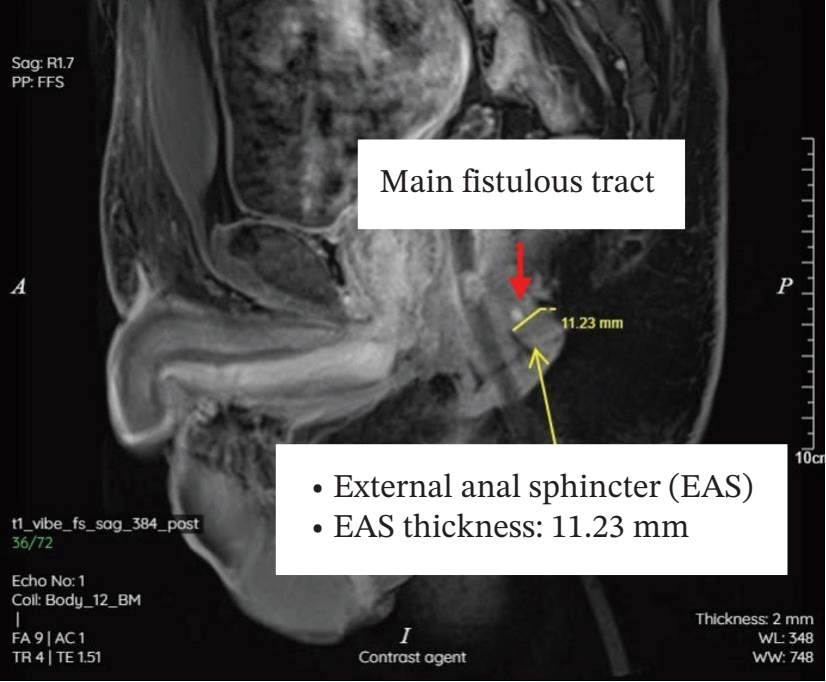
Preoperative sagittal MRI demonstrating the main fistulous tract traversing the external anal sphincter (EAS), with an EAS thickness of 11.23 mm as measured on MRI.

Based on the MRI findings, the fistula was classified as Garg Grade 1, Parks Grade II (transsphincteric), and St. James’s University Hospital MRI Grade III [9, 10, 12].

The surgery was performed under spinal anesthesia with the patient in the lithotomy position. Preoperative MRI findings were carefully reviewed to identify the expected location of the internal opening. During examination under anesthesia, careful inspection together with digital palpation revealed inflammatory changes and subtle tissue retraction suggestive of the posterior internal opening. Hydrogen peroxide was then gently injected through the external opening. White foaming bubbles emerged from the posterior midline internal opening, thereby confirming the MRI findings before definitive surgical treatment (Figure 6).

**Figure 6:**
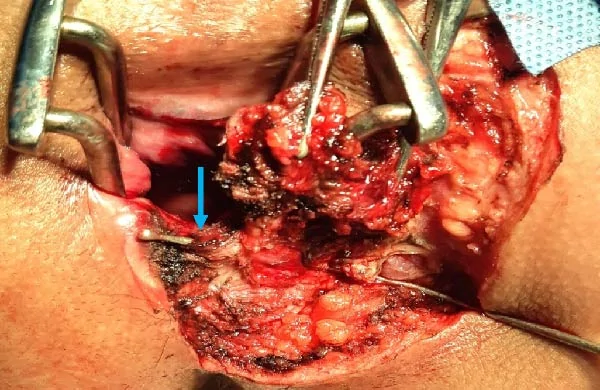
Intraoperative photograph demonstrating the transsphincteric portion of the fistulous tract (blue arrow) crossing the external anal sphincter, consistent with the preoperative MRI findings.

The extrasphincteric portion of the fistulous tract, together with the surrounding chronically inflamed and fibrotic tissue, was excised (partial fistulectomy), whereas the transsphincteric portion traversing the external anal sphincter was treated by fistulotomy (lay‐open technique) to preserve sphincter function (Figure 7A). No tissue biopsy was performed because there was no clinical suspicion of malignancy, Crohn’s disease, or other specific pathology requiring a histopathological examination.

**Figure 7:**
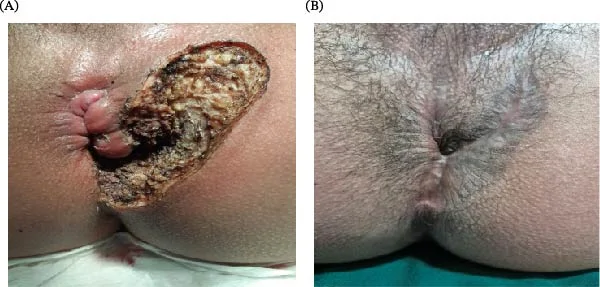
Surgical outcome. (A) Immediate postoperative appearance after combined fistulectomy of the extrasphincteric segment of the tract and lay‐open (fistulotomy) of the transsphincteric portion. The wound was left open for secondary healing. (B) Follow‐up photograph at 2 months demonstrating complete epithelialization without deformity of the anal canal.

The wound was left completely open for healing by secondary intention without marsupialization. Standard postoperative wound care, consisting of daily irrigation and dressing changes, was performed. Complete wound healing was achieved within 2 months (Figure 7B). At the 12‐month follow‐up, the patient remained asymptomatic, with no evidence of fistula recurrence or fecal incontinence.

## 3. Discussion

Accurate identification of the internal opening remains a cornerstone of successful anal fistula surgery because failure to correctly identify the primary tract and internal opening may result in persistent sepsis, recurrence, or unnecessary sphincter division with subsequent impairment of continence [1, 2]. In the present case, the external opening was located at the 2 o’clock position and more than 3 cm from the anal verge. Preoperative MRI demonstrated a curvilinear tract extending posteriorly to a posterior midline internal opening. This anatomical course was subsequently confirmed by examination under anesthesia and hydrogen peroxide injection, allowing accurate operative planning.

Goodsall’s rule has served for more than a century as a practical clinical guide for predicting the likely course of an anal fistula and the location of its internal opening according to the position of the external opening [3, 4]. In the present case, the anterior external opening located more than 3 cm from the anal verge was consistent with the classical description of a curvilinear tract toward the posterior midline. Thus, Goodsall’s rule provided an initial clinical orientation rather than a definitive determination of the fistulous anatomy. Contemporary studies have demonstrated that the predictive accuracy of Goodsall’s rule is variable and influenced by fistula anatomy and complexity [5–7]. Therefore, it should be regarded as a useful clinical guide rather than an absolute anatomical rule. In this case, MRI was used to delineate the complete fistulous anatomy and to determine the relationship of the tract to the external anal sphincter.

MRI is an important imaging modality for the preoperative evaluation of anal fistulas because it delineates the primary tract, identifies the internal opening, assesses sphincter involvement, and detects secondary extensions or occult abscesses that may influence surgical planning [2, 9–11]. In the present case, MRI not only confirmed the posterior midline internal opening but also demonstrated that the external anal sphincter measured ~11.23 mm in thickness and that the main fistulous tract traversed ~30% of its thickness. These findings were important in determining the extent of sphincter involvement and in selecting an appropriate surgical strategy. The complete concordance between MRI and intraoperative findings further supported the reliability of the preoperative anatomical assessment.

The surgical approach in this case was individualized according to the anatomical relationship of each component of the fistulous tract to the external anal sphincter. The extrasphincteric segment of the fistulous tract, extending from the external opening to the external border of the external anal sphincter, was excised together with the surrounding chronically inflamed and fibrotic tissue until healthy tissue was reached. Because this component was located outside the sphincter complex, its excision did not require additional division of the sphincter muscle. In contrast, the transsphincteric component was classified as Parks Grade II and Garg Grade I (low, single tract). Given that the main tract was estimated on MRI to traverse ~30% of the external anal sphincter thickness, we deliberately avoided circumferential excision of the tissue surrounding the transsphincteric tract and instead performed a lay‐open fistulotomy. This segment‐specific approach was intended to provide adequate treatment of the fistulous tract while minimizing unnecessary sacrifice of the sphincter tissue.

The choice between fistulotomy and fistulectomy remains an area of clinical discussion, particularly regarding healing, recurrence, and preservation of continence. A recent systematic review and meta‐analysis of 13 randomized controlled trials involving 1373 patients with simple fistula‐in‐ano found no statistically significant difference between fistulotomy and fistulectomy in recurrence, fecal incontinence, wound infection, or healing time [13]. According to the Parks classification, the fistula in the present case was classified as Grade II (transsphincteric) [14]. These findings do not establish a superiority of either technique for simple fistulas. However, they should not be directly extrapolated to all transsphincteric fistulas, particularly those with greater sphincter involvement or a more complex anatomy. In the present case, the decision to perform fistulotomy for the transsphincteric component was based on the limited sphincter involvement demonstrated by MRI and the objective of preserving as much functional sphincter tissue as reasonably possible. We do not suggest that transsphincteric fistulectomy is inappropriate in general; rather, our approach was individualized to the anatomy of this particular low, single‐tract fistula.

The present case also illustrates the importance of integrating clinical assessment, imaging, and intraoperative findings rather than relying on a single diagnostic method. Goodsall’s rule provided an initial clinical orientation, MRI provided detailed anatomical mapping and assessment of sphincter involvement, and intraoperative examination with hydrogen peroxide confirmed the internal opening. The combination of these approaches allowed a segment‐specific surgical strategy that balanced the treatment of the fistulous tract with the preservation of sphincter function. At 12 months, the patient remained asymptomatic without recurrence or fecal incontinence. However, as this is a single case report, the favorable outcome cannot establish the superiority of this surgical approach or demonstrate a reduction in recurrence risk.

This case illustrates the complementary roles of clinical assessment, Goodsall’s rule, MRI, and intraoperative confirmation in accurately localizing the internal opening of a cryptoglandular anal fistula. Goodsall’s rule provided an initial clinical orientation, while MRI precisely delineated the fistulous anatomy and its relationship to the external anal sphincter, thereby facilitating an anatomy‐based and sphincter‐preserving surgical strategy. In this patient, the extrasphincteric component was treated by fistulectomy, whereas the low, single‐tract transsphincteric component was treated by lay‐open fistulotomy. Although the favorable outcome in this individual patient supports the feasibility of this approach, a single case cannot establish its superiority or its effect on recurrence. The prevalence of anal fistula has been investigated in a systematic review and meta‐analysis [15].

## Funding

No funding was received for this manuscript.

## Ethics Statement

Ethical approval was waived by the Medical Ethics Committee of Dong A University for this single case report.

## Consent

Written informed consent was obtained from the patient for publication of this case report and accompanying images.

## Conflicts of Interest

The author declares no conflicts of interest.

## Data Availability

The data supporting the findings of this study are available within the article.

## References

[1] Gaertner W. B. , Burgess P. L. , and Davids J. S. , et al.The American Society of Colon and Rectal Surgeons Clinical Practice Guidelines for the Management of Anorectal Abscess, Fistula-in-Ano, and Rectovaginal Fistula, Diseases of the Colon and Rectum. (2022) 65, no. 8, 964–985, 10.1097/DCR.0000000000002473. 35732009

[2] Reza L. , Göttgens K. , and Kleijnen J. , et al.European Society of Coloproctology: Guidelines for Diagnosis and Treatment of Cryptoglandular Anal Fistula, Colorectal Disease. (2024) 26, no. 1, 145–196, 10.1111/codi.16741. 38050857

[3] Goodsall D. H. , Diseases of the Anus and Rectum, 1900, Longmans, Green.

[4] Parks A. G. , Pathogenesis and Treatment of Fistula-in-Ano, BMJ. (1961) 1, no. 5224, 463–460, 10.1136/bmj.1.5224.463. PMC1953161 13732880

[5] Devi V. S. , Thulasibai S. K. L. , and Deepak P. , et al.Goodsall’s Rule—Its Predictive Accuracy in Tracing the Tract of Fistula in Ano, International Surgery Journal. (2020) 7, no. 12, 4116–4119, 10.18203/2349-2902.isj20204955.

[6] Garcia-Bottello S. , Martin-Arevalo J. , and Marti-Fernandez R. , et al.Three-Dimensional Endoanal Ultrasound to Assess the Validity of Goodsall’s Rule and the Midline Rule in Predicting the Path of Perianal Fistulas and the Location of the Internal Opening, Techniques in Coloproctology. (2022) 26, no. 5, 351–361, 10.1007/s10151-022-02592-2. 35217938

[7] Bakir Q. K. , Noori I. F. , and Noori A. F. , Accuracy Prediction of Goodsall’s Rule for Anal Fistulas of Cryptoglandular Origin: Is It Still Standing?, Annals of Medicine & Surgery. (2024) 86, no. 5, 2453–2457, 10.1097/MS9.0000000000001554. PMC11060315 38694374

[8] Öz D. K. , Zorlu S. N. Y. , and Eskalen Z. , et al.Goodsall’s Rule Revisited: An MRI-Based Assessment of its Accuracy in Perianal Fistulas, Turkish Journal of Colorectal Disease. (2025) 35, no. 2, 41–47, 10.4274/tjcd.galenos.2025.2025-1-2.

[9] Morris J. , Spencer J. A. , and Ambrose N. S. , MR Imaging Classification of Perianal Fistulas and Its Implications for Patient Management, RadioGraphics. (2000) 20, no. 3, 623–635, 10.1148/radiographics.20.3.g00mc15623. 10835116

[10] de Miguel Criado J. , García del Salto L. , and Fraga Rivas P. , et al.MR Imaging Evaluation of Perianal Fistulas: Spectrum of Imaging Features, RadioGraphics. (2012) 32, no. 1, 175–194, 10.1148/rg.321115040. 22236900

[11] Sudoł-Szopińska I. , Garg P. , and Mellgren A. , et al.Structured Magnetic Resonance Imaging and Endoanal Ultrasound Anal Fistulas Reporting Template (SMART): An Interdisciplinary Delphi Consensus, World Journal of Gastrointestinal Surgery. (2024) 16, no. 10, 3288–3300, 10.4240/wjgs.v16.i10.3288. PMC11577389 39575264

[12] Garg P. , Garg Classification of Fistula-in-Ano: Rationale and Surgical Outcomes, Colorectal Disease. (2017) 19, no. 1, 17–22.

[13] Quinn R. , Peacock A. , and Lendzion R. J. , Fistulotomy Versus Fistulectomy for Simple Fistula-in-Ano: A Systematic Review and Meta-Analysis of Randomized Controlled Trials, ANZ Journal of Surgery. (2025) 95, no. 6, 1074–1090, 10.1111/ans.70095. PMC12227843 40125894

[14] Parks A. G. , Gordon P. H. , and Hardcastle J. D. , A Classification of Fistula-in-Ano, Journal of British Surgery. (1976) 63, no. 1, 1–12, 10.1002/bjs.1800630102. 1267867

[15] Sarveazad A. , Bahardoust M. , Shamseddin J. , and Yousefifard M. , Prevalence of Anal Fistulas: A Systematic Review and Meta-Analysis, Gastroenterology and Hepatology From Bed to Bench. (2022) 15, no. 1, 1–8. PMC9123633 35611255

